# Polyampholytic Graft Copolymers as Matrix for TiO_2_/Eosin Y/[Mo_3_S_13_]^2−^ Hybrid Materials and Light‐Driven Catalysis

**DOI:** 10.1002/chem.202100091

**Published:** 2021-03-08

**Authors:** Afshin Nabiyan, Johannes Bernhard Max, Christof Neumann, Magdalena Heiland, Andrey Turchanin, Carsten Streb, Felix Helmut Schacher

**Affiliations:** ^1^ Institute of Organic Chemistry and Macromolecular Chemistry (IOMC) Friedrich Schiller University Jena Lessingstraße 8 07743 Jena Germany; ^2^ Jena Center for Soft Matter (JCSM) Friedrich Schiller University Jena Philosophenweg 7 07743 Jena Germany; ^3^ Center for Energy and Environmental Chemistry Jena (CEEC Jena) Friedrich Schiller University Jena Philosophenweg 7a 07743 Jena Germany; ^4^ Institute of Physical Chemistry and Abbe Center of Photonics Friedrich Schiller University Jena Lessingstr. 10 07743 Jena Germany; ^5^ Institute of Inorganic Chemistry I Ulm University Albert-Einstein-Allee 11 89081 Ulm Germany

**Keywords:** hybrid materials, hydrogen evolution reaction, photocatalysis, polyampholytes, thiomolybdates

## Abstract

An effective strategy to enhance the performance of inorganic semiconductors is moving towards organic‐inorganic hybrid materials. Here, we report the design of core–shell hybrid materials based on a TiO_2_ core functionalized with a polyampholytic (poly(dehydroalanine)‐*graft*‐(*n*‐propyl phosphonic acid acrylamide) shell (PDha‐*g*‐PAA@TiO_2_). The PDha‐*g*‐PAA shell facilitates the efficient immobilization of the photosensitizer Eosin Y (EY) and enables electronic interactions between EY and the TiO_2_ core. This resulted in high visible‐light‐driven H_2_ generation. The enhanced light‐driven catalytic activity is attributed to the unique core–shell design with the graft copolymer acting as bridge and facilitating electron and proton transfer, thereby also preventing the degradation of EY. Further catalytic enhancement of PDha‐*g*‐PAA@TiO_2_ was possible by introducing [Mo_3_S_13_]^2−^ cluster anions as hydrogen‐evolution cocatalyst. This novel design approach is an example for a multi‐component system in which reactivity can in future be independently tuned by selection of the desired molecular or polymeric species.

## Graphical abstract

**A tailor‐made polyampholyte matrix** enables effective interaction of different components in light‐driven hydrogen evolution reaction, in our case TiO_2_ nanoparticles, thiomolybdate [Mo_3_S_13_]^2−^ clusters, and Eosin Y as photosensitizer. Altogether, high photocatalytic activity for H_2_ generation under visible‐light irradiation was observed for well‐defined hybrid materials.

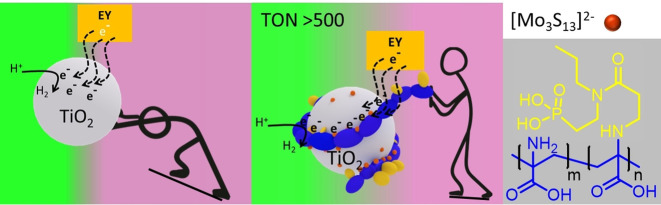

## Introduction

Global challenges such as climate change and overconsumption urgently require the supply of sustainable clean energy. This could be tackled by the utilization of hydrogen as a secondary, carbon‐free energy carrier. Therefore, tremendous efforts have been invested in the direction of production, storage, and delivery of hydrogen.^[1]^ Light‐driven catalytic hydrogen evolution is a favourable carbon‐neutral method, exploiting solar energy.^[2]^ The first example of light‐induced water splitting was described in 1972 by utilisation of a TiO_2_ electrode, though it was limited to irradiation with UV light.^[3]^ Although being chemically stable, non‐toxic, and a low cost material, light harvesting using a broader solar energy spectrum is desirable.^[4]^ Recent approaches towards hydrogen evolution are therefore targeting visible‐light harvesting through band gap engineering,^[5]^ utilization of photosensitizers,^[6]^ introduction of co‐catalysts,^[7]^ electron relays such as polyoxometalates,^[8]^ or the use of novel semi‐conducting materials.^[9]^ In this context, TiO_2_ as potential candidate can be tuned for visible‐light‐driven hydrogen evolution by the addition of different cocatalysts and sensitizers. Recently, combining TiO_2_ and molybdenum sulfide was identified as a promising composite system.[^4c^, ^10^]

Molybdenum sulfides (MoS_*x*_) as (co)catalysts are gaining considerable interest due to low cost, long‐term stability, and earth‐abundance. In that regard both, 2D structured MoS_2+*x*_ as well as molecular molybdenum sulfides (thiomolybdates), for example, [Mo_3_S_13_]^2−^ or [Mo_2_S_12_]^2−^ have been investigated in combination with different sensitizers and (co)catalysts.[^4b^, ^11^] Thiomolybdates typically carry a high number of active sites and allow homogenous hydrogen evolution with high turnover numbers (>41 000).[^11b^, ^11c^, ^12^]

With respect to multi‐component hybrid materials, immobilization of different compounds within suitable matrices plays a key role in connecting individual elements of light‐driven catalytic systems and has been realized on solid substrates such as carbon nanomaterials,^[13]^ metal oxides^[14]^ or semiconductors such as p‐Si.^[15]^ Although being well soluble in aqueous environment and exhibiting potential binding sites for both different catalysts and sensitizers, only few examples focus on soft matrices based on polyelectrolytes. Romanenko et al. described the preparation of block copolymer membranes, where the molecular catalyst [Mo_3_S_13_]^2−^ and photosensitizer [Ru(bpy)_3_]^2+^ were immobilized using positively charged groups along the poly(*N,N*‐dimethylaminoethyl methacrylate) (PDMAEMA) segment.^[16]^ Besides, polyelectrolyte‐based hydrogels are promising scaffolds and in this regard Weingarten et al.^[17]^ and H. Sai et al.^[18]^ attached perylene monoimide as well as suitable catalysts for efficient hydrogen production. Also ‘free’ polymers could molecularly interact with catalysts as it has been shown by Hu et al., exploiting conjugated polyelectrolytes for the interaction with Pt catalysts in hydrogen evolution reactions,^[19]^ or double‐hydrophilic block copolymers as templates for CdS nanoparticles.^[20]^

Polyampholytic polydehydroalanine (PDha) is a suitable template featuring a high density of functional (charged) groups and strongly interacting with metal oxides, metal nanoparticles and dyes in water, which led us to the assumption that this is a promising matrix for light‐driven catalysis.^[21]^ PDha exhibits both positively charged amino groups as well as negatively charged carboxylic acid moieties for the specific interaction with various compounds. Furthermore, it was found to be a platform for modification reactions to obtain materials with tailored solubility, for example, as sensors, smart dispersants, or templates.[^21c^, ^22^] Besides this, PDha‐*g*‐PEG was already successfully used as dispersant for a water‐insoluble perylene‐based photosensitizer.^[23]^ In this regard, we prepared tailor‐made PDha graft copolymers for the application in visible‐light driven hydrogen evolution. We herein introduce a novel catalytic system consisting of promising and low cost catalysts TiO_2_ and [Mo_3_S_13_]^2−^, as well as Eosin Y (EY) as dye sensitizer in the presence of the sacrificial agent triethanol amine (TEOA), revealing >800 fold increased activity compared to bare EY/TiO_2_ hybrids. This is realized by the attachment of phosphonic acid side‐chains for increased solution stability against sedimentation and as strong anchor groups for TiO_2_.^[15]^ The overall combination not only leads to visible light‐driven catalysis, but also enables the physical combination of materials, which has not been possible before. The mere combination of TiO_2_ and EY has already been reported, but efficient hydrogen evolution strongly depends on the way of interaction due to insulating, quenching and stability issues.[^1c^, ^24^] On the other hand, while EY was successfully used as a photosensitizer together with MoS_2,_ ^[25]^ it failed in case of [Mo_3_S_13_]^2−^ presumably due to rather weak interactions with the catalyst.^[11b]^

## Results and Discussion

### Synthesis of phosphonic acid modified graft copolymers

**Figure 1:**
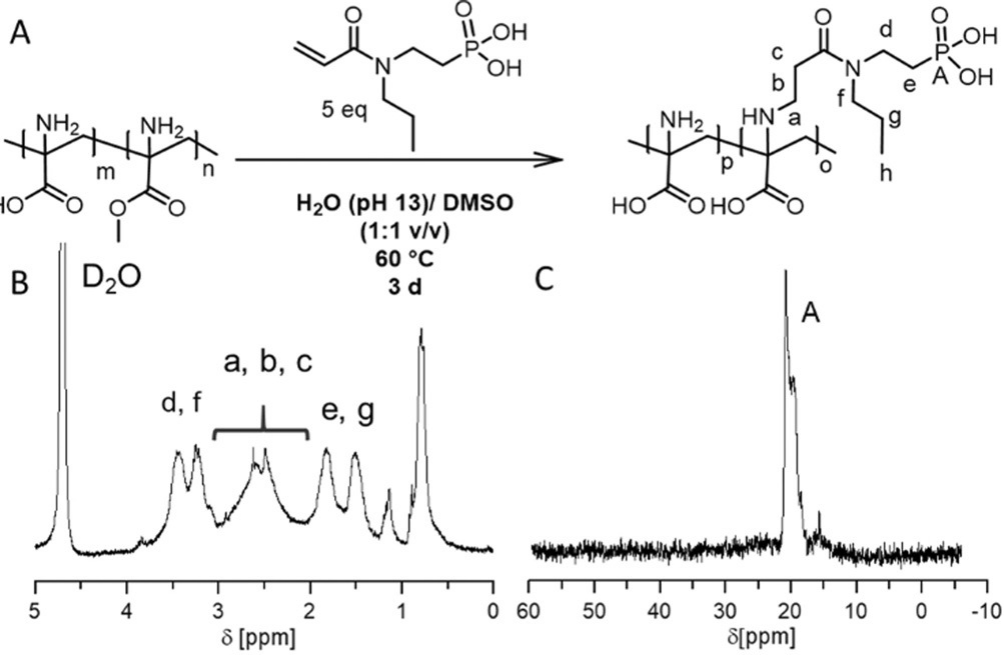
Synthesis of PDha‐*g*‐PAA graft copolymers (A) and corresponding ^1^H‐NMR (B) and ^31^P‐NMR (C) spectra.

In our earlier work we have shown that post‐polymerization modification of PDha by grafting is a powerful synthetic route to fine‐tune solution properties and functionality.[^21c^, ^22a^] To develop the applicability of PDha as a coating for TiO_2_ NPs, both increasing its water solubility at pH values <7 and the attachment of an additional, strong anchor group was desired. Therefore, PDha with an average of 60 repeat units was used as a reactive backbone, obtained from deprotection of P*t*BAMA (M_n_=13 200, Đ=2.55), and *n*‐propyl phosphonic acid acrylamide (PAA) was successfully grafted via an aza‐Michael addition (Figure 1 **A**).

The modifier was synthesized in accordance with the protocol of Hu et al.^[26]^ Hereby, 66 % of the monomer units were functionalized when 5 equivalents of the acryl amide were used. The degree of functionalization was determined from ^1^H NMR spectroscopy in accordance with our previous work.^[22a]^ As expected, the obtained graft copolymer was soluble over the entire pH‐range as well as in methanol. NMR (^31^P, ^1^H and ^13^C) spectroscopy (Figure 1 A, B; Figure S2) shows the presence of both the PDha backbone as well as phosphonic acid side groups and SEC traces reveal a narrowing of the elution traces in comparison to the pristine polymer, while still maintaining a monomodal distribution. Regarding the multiple peaks in the ^31^P NMR spectrum being in close proximity, we assume a random distribution of the side‐chains along the backbone leading to different chemical environments of the phosphonic acid groups. Besides that, protonation might also play a role.

### Formation of TiO_2_ based core–shell hybrids

**Figure 2:**
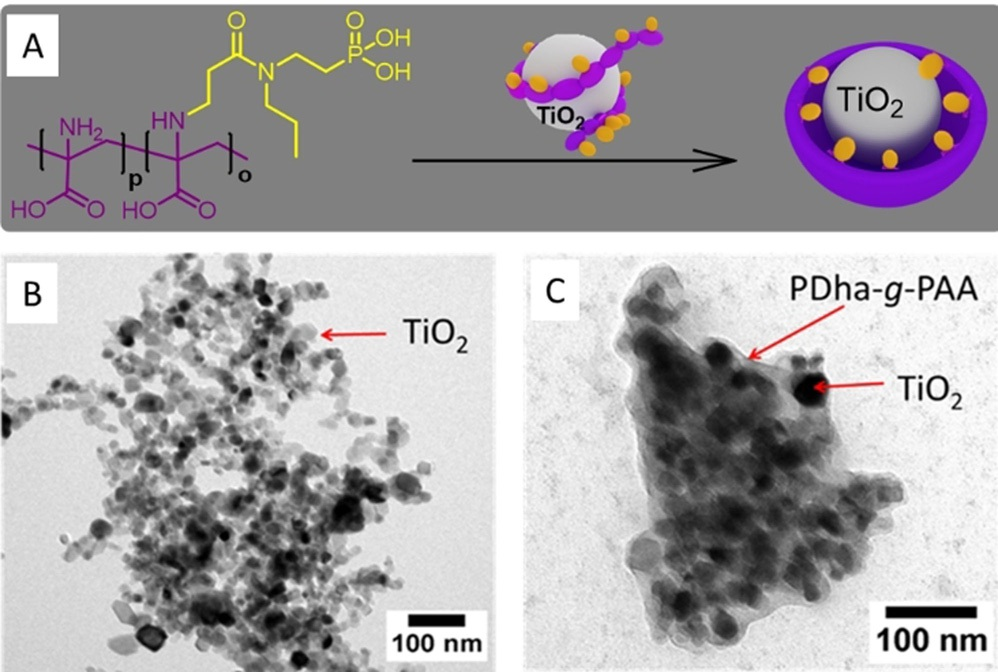
Preparation of PDha‐*g*‐PAA@TiO_2_ core–shell hybrid materials (A), TEM images of TiO_2_ (B) and PDha‐*g*‐PAA@TiO_2_ with 15:1 (w^/^w, polymer/TiO_2_) as initial ratio of PDha‐*g*‐PAA/TiO_2_.

The amino and carboxylic acid moieties of PDha are exceptionally strong ligands for inorganic NPs as already proven for stable dispersions with iron oxide, gold, silver, or Ag/Au nanoalloy particles.[^21a^, ^21c^, ^27^] However, the readily grafted phosphonic acid side‐chains are additional strong ligands for TiO_2_ surfaces and besides that they implement pH solution stability as well as additional negative charges, the latter possibly facilitating proton transfer processes.^[28]^ Therefore, we utilized PDha‐*g*‐PAA as coating for TiO_2_ NPs using simple ultra‐sonication (Figure 2 A) and the successful formation of core–shell hybrid materials was proven by thermogravimetric analysis (TGA), transmission electron microscopy (TEM), X‐ray photoelectron spectroscopy (XPS) and dynamic light scattering (DLS) (Figures S3–S6).

To determine the amount of organic shell material, TGA was measured under air for the pristine TiO_2_, PDha‐*g*‐PAA and the hybrid materials with different polymer to TiO_2_ ratios (5:1 and 15:1 (w/w) polymer/TiO_2_, Figure S3). Any free polymer was removed by dialysis and three washing steps after centrifugation of the solid material. For the TiO_2_ NPs a weight loss of around 17 wt % was observed between 30 °C and 450 °C, which is mainly assigned to the loss of water. In case of PDha‐*g*‐PAA three weight‐loss steps were observed, the first step between rt and 140 °C as a result of loss of residual water, and two further steps at 290 and 470 °C as a result of polymer decomposition. No further weight loss is observed at *T*>600 °C (residual mass: 20 wt %). Regarding the hybrid material, similar decomposition steps were observed between 290 and 600 °C, providing evidence for the presence of the polymer shell. High polymer contents of 29 wt % and 66 wt % were calculated, respectively. To confirm these results, TEM images of PDha‐*g*‐PAA@TiO_2_ (Figure 2 and Figures S4 and S5) show individual TiO_2_ NPs with an average radius of around 11 nm and an organic layer with a thickness of several nm is clearly visible. Additionally, in DLS an increase of the hydrodynamic radius (*R*_H_) from 11 to 38 nm was observed after shell formation (Figure S3). We attribute this significant increase in size to multilayer formation and chain expansion of the grafted polymers.

### [Mo_3_S_13_]^2−^ as cocatalyst modification

**Figure 3:**
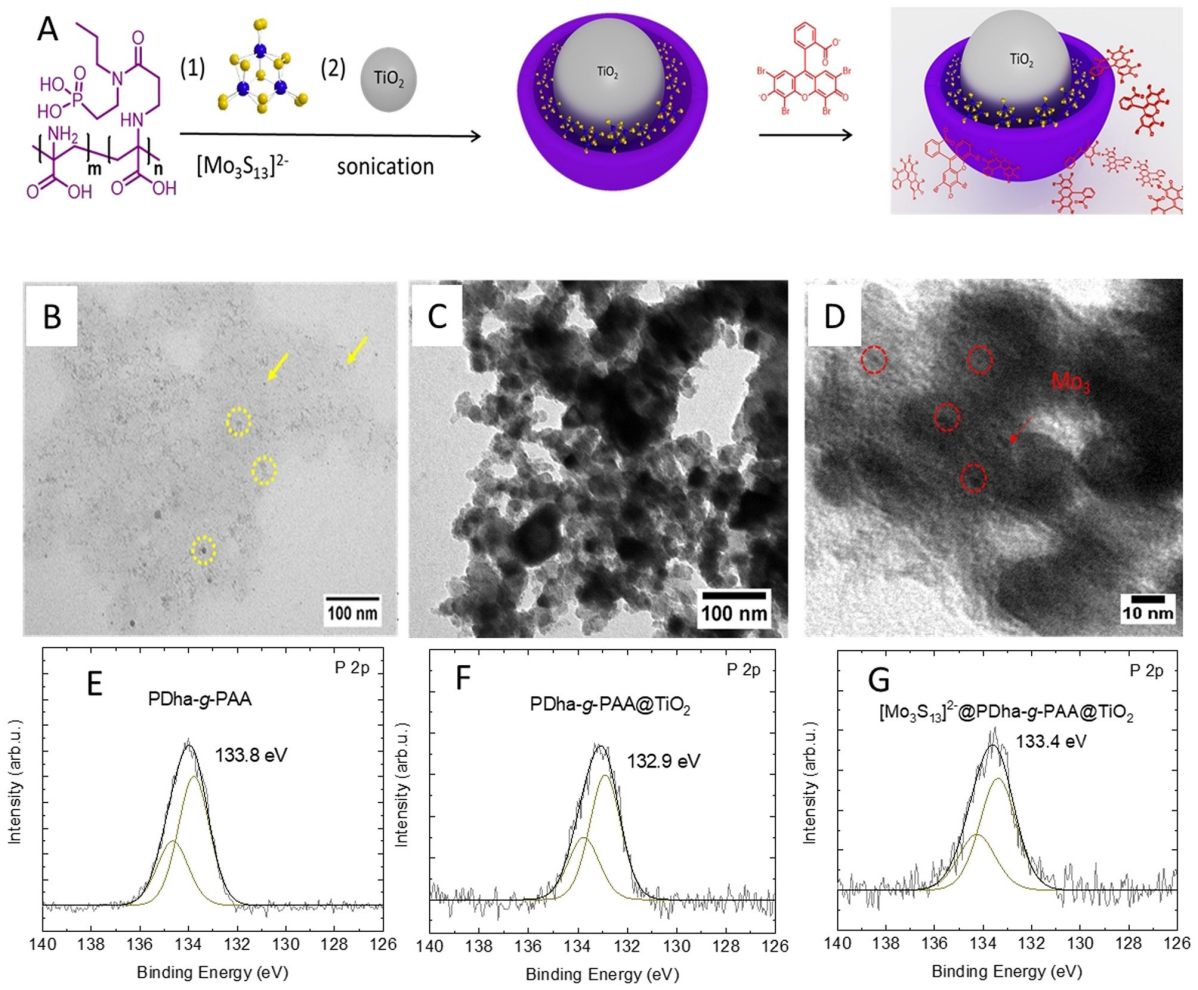
(A): Preparation route of [Mo_3_S_13_]^2−^@PDha‐*g*‐PAA@TiO_2_, TEM images of [Mo_3_S_13_]^2−^@PDha‐*g*‐PAA (B), and (C and D): [Mo_3_S_13_]^2−^@PDha‐*g*‐PAA@TiO_2_^,^and (E–G): XP spectra of P 2p of PAA in PDha‐*g*‐PAA (E), PDha‐*g*‐PAA@TiO_2_ (F), and (G) [Mo_3_S_13_]^2−^@PDha‐*g*‐PAA@TiO_2_.

To further overcome the recombination of photoexcited carriers, cocatalyst modification was explored for the PDha‐*g*‐PAA@TiO_2_ core–shell hybrid system. Thereby, as a co‐catalyst we used [Mo_3_S_13_]^2−^ clusters in accordance with our earlier work.^[16]^ Hereby, PDha‐*g*‐PAA on one hand solubilizes negatively charged molybdenum sulfide, and on the other hand is anchored on the surface of the TiO_2_ nanoparticles (Figure 3 A), therefore bringing both building blocks in close proximity while at the same time mediating solubility.

The resulting [Mo_3_S_13_]^2−^@PDha‐*g*‐PAA and [Mo_3_S_13_]^2−^@PDha‐*g*‐PAA@TiO_2_ hybrid materials were characterized via DLS and TEM (Figure 3 and Figure S3, S4). TEM data indicates small aggregates with the size of only a few nm for [Mo_3_S_13_]^2−^@PDha‐*g*‐PAA (Figure 3 B). After successful solubilisation of [Mo_3_S_13_]^2−^_,_ TiO_2_ NPs were added before further sonication and the resulting three‐component hybrid material ([Mo_3_S_13_]^2−^@PDha‐*g*‐PAA_15_@TiO_2_) was subjected to TEM investigations (Figure 3 C, D and Figure S9), where in addition to the above‐described core–shell nanoobjects additional dark spots with different contrast could be found. We ascribe these likely to the presence of the co‐catalyst as schematically shown in Figure 3 A and Figure S8. However, DLS reveals a significant increase of the *R*_H_ of the three‐component hybrid material compared to PDha‐*g*‐PAA@TiO_2_ (15 w/w) from 38 to 82 nm, which could be the result of multilayer formation, chain expansion, and some secondary aggregation taking place.

In addition, ζ‐potential measurements were carried out and while the overall charge of the TiO_2_ NP was found to be slightly negative with −8±2 mV, it decreased to −20±1 after addition of the graft copolymer. We ascribe this to the negatively charged carboxylic acid and phosphonic acid groups. After decoration of the core–shell material with anionic [Mo_3_S_13_]^2−^ the ζ‐potential became −32 ± and finally −37±1 after attachment of EY, indicating successive incorporation of the different compounds. This is noteworthy as electrostatic repulsion might also be expected—nevertheless, additional (attractive) interactions, for example, with exposed ‐NH_3_^+^ functional groups from the PDha backbone or of hydrophobic nature seem to favor binding of the individual components. Indeed, the amine groups of the polyampholytic backbone should be protonated at the corresponding pH of 7–8, although an overall negative charge is observed.[^21c^, ^22a^, ^29^]

In order to further investigate the interactions between PDha‐*g*‐PAA with [Mo_3_S_13_]^2−^ and TiO_2_, X‐ray photoelectron spectroscopy (XPS) was used. The C 1s, P 2p, Mo 3d, Ti 2p, N 1s, and O 1s spectra obtained by XPS for different samples are shown in Figure 3 E–G and Figures S5, S6. In the Ti 2p spectra, the doublet was assigned to TiO_2_, with Ti 2p_3/2_ at a binding energy of ≈459 eV and Ti 2p_1/2_ at a binding energy of ≈464.5 eV. The binding energy difference of 5.5 eV between those two peaks of TiO_2_ corresponds well to literature values (Δ*E* from 5.5 to 5.8 eV).^[30]^ Following grafting of PDha‐*g*‐PAA, no significant changes are observed in the Ti 2p spectrum. After grafting of the polymer, a P 2p signal was detected, which was not observed for the TiO_2_ reference particles. This confirms the presence of the phosphonic acids on the modified TiO_2_ particles. Interestingly, the binding energy of phosphonic acid is shifted towards lower binding energies upon grafting onto TiO_2_ from 133.8 eV for free PDha‐*g*‐PAA to 132.9 and 133.4 eV for PDha‐*g*‐PAA@TiO_2_ and [Mo_3_S_13_]^2−^@PDha‐*g*‐PAA@TiO_2_, respectively (Figure 3 E **–**G). The observed downshift is another indication for successful grafting using phosphonic acid as anchoring group.[^30a^, ^31^]

### Visible‐light driven hydrogen evolution

**Figure 4:**
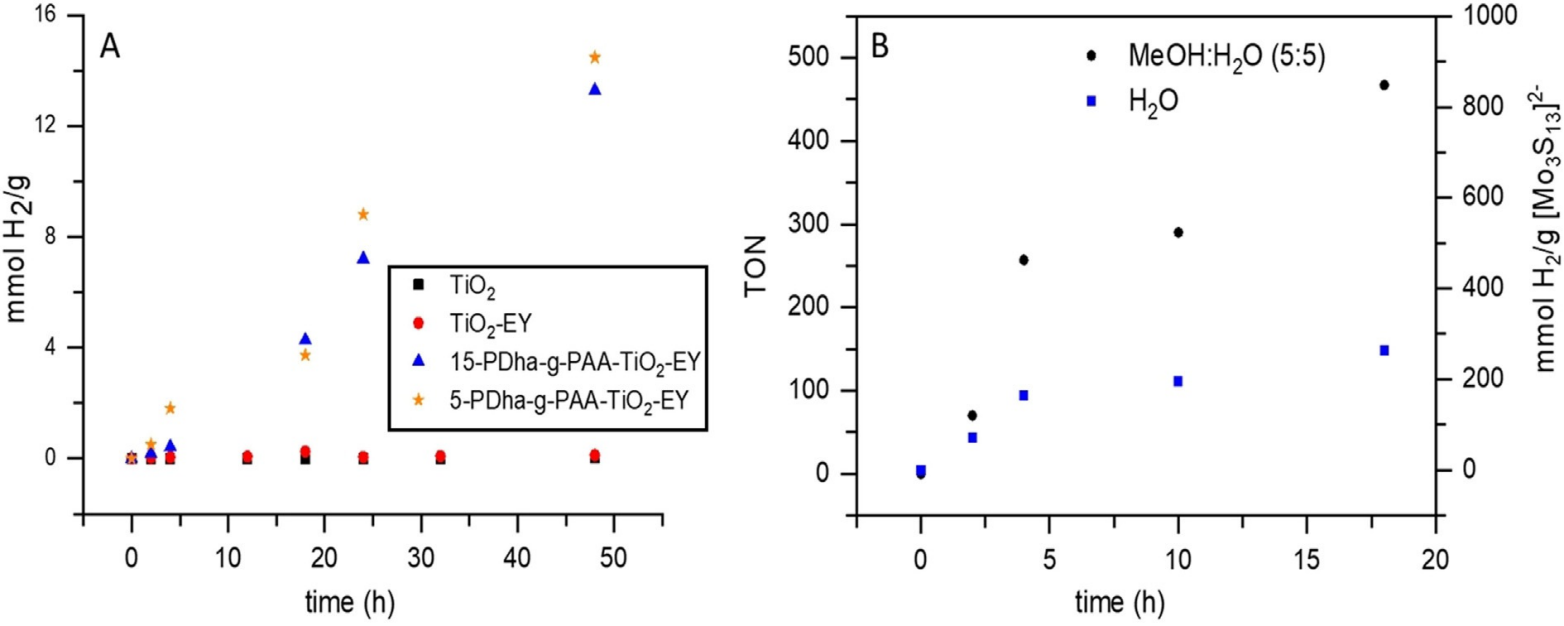
Hydrogen evolution rates for (A): TiO_2_ (300.0 μmol L^−1^), TiO_2_/EY (20.0 μmol), and EY/PDha‐*g*‐PAA/TiO_2_ with different initial weight percent (5 and 15 w/w) of PDha‐*g*‐PAA to TiO_2_ in water and (B): Hydrogen evolution rates and the turnover number (TON, is defined as moles of hydrogen produced to moles of [Mo_3_S_13_]^2−^ (3 μmol L^−1^)) of EY/PDha‐*g*‐PAA/(TiO_2_/[Mo_3_S_13_]^2−^) with 5:1 w/w initial weight (PDha‐*g*‐PAA to TiO_2_) in water and mixture of water/methanol under visible‐light irradiation (λ>520 nm), with TEOA (0.5 m) as sacrificial donor.

Light‐driven catalytic H_2_ evolution performances of the samples were evaluated in the presence of TEOA (0.5 m) as sacrificial reagent under LED light irradiation (530 nm (with ±50 nm; 281 mW; 330 mA; 3.1 V) in a 3D‐printed irradiation reactor with a fan (Figure S1). To investigate the effect of each compound of our system different tests were made. Figure 4 A shows the time courses of H_2_ production for TiO_2_, TiO_2_/EY, and EY/PDha‐g‐PAA/TiO_2_ with different weight percent (wt %) of polymer. TiO_2_ without EY and physical mixtures of TiO_2_ with EY show no or trace H_2_ evolution activity under these conditions. We could explain this by inactivity of TiO_2_ under visible light irradiation and the lack of firmly bound EY to the TiO_2_ surface.^[32]^ Besides low loading of TiO_2_ via a weak ester‐like linkage, physically adsorbed dyes tend to desorb into the solution during irradiation, leading to quick degradation due to the formation of unstable anion radicals (EY^−.^), and decreasing efficiency in H_2_ evolution catalysis. In sharp contrast, coating TiO_2_ with PDha‐*g*‐PAA significantly increased the H_2_ production under visible light. Overall, during irradiation, EY/PDha‐*g*‐PAA/TiO_2_ with 5:1 and 15:1 of PDha‐*g*‐PAA to TiO_2_ (w/w) exhibits high stability for hydrogen production with average rates of 0.301 and 0.276 mmol g^−1^ h^−1^, respectively.

The enhanced activity of EY/PDha‐*g*‐PAA/TiO_2_ is attributed to an effective EY loading by PDha‐*g*‐PAA and stable fixation of EY within the polyampholyte shell. The maximum loading amount of EY by PDha‐*g*‐PAA and TiO_2_ NPs was evaluated by a UV/Vis study (Figure S7 and Table S1). Our data revealed capacity values of 151 μg mg^−1^ for TiO_2_, and 102 and 76 μg mg^−1^ for EY/PDha‐*g*‐PAA/TiO_2_ with 5:1 and 15:1 (w/w) of graft copolymer, respectively. The maximum loading capacity for the EY/PDha‐*g*‐PAA/TiO_2_ coating material resembled the capacity of PDha‐*g*‐PAA as well, whereas it was slightly lower compared to pristine TiO_2_, probably due to the rather high surface area of TiO_2_. Nevertheless, this might also enhance the fast decomposition of dye during irradiation, leading to lower hydrogen evaluation rates. On the other hand, although PDha‐*g*‐PAA/TiO_2_ shows lower capacity for EY adsorption, it is seemingly enough adsorbed and stable to inject the electron though the graft copolymer shell to the TiO_2_ core.

For improving the performance of PDha‐*g*‐PAA/TiO_2_ core–shell hybrids, additional loading with thiomolybdate [Mo_3_S_13_]^2−^ nanoclusters as a molecular mimic of MoS_2_ edge sites was investigated. Hereby, PDha‐*g*‐PAA was used first to solubilize [Mo_3_S_13_]^2−^ and afterwards PDha‐*g*‐PAA/[Mo_3_S_13_]^2−^ was grafted onto TiO_2_ NPs as described above (Figure 3 A). However, EY/TiO_2_/[Mo_3_S_13_]^2−^ were also physically mixed as control experiment and here no hydrogen evolution could be observed under the conditions reported. In contrast, under optimum reaction conditions for the EY/PDha‐*g*‐PAA/(TiO_2_/[Mo_3_S_13_]^2−^) system, the H_2_ production under visible light irradiation was continuous during 20 h of reaction time. The H_2_ evolution turnover number (TON=[H_2_]/[Mo_3_S_13_]^2−^]) reaches >500 within 20 h of irradiation. As we can see from H_2_ production data, the activities of H_2_ production from EY/PDha‐*g*‐PAA/(TiO_2_/[Mo_3_S_13_]^2−^) system are significantly improved compared to the system without [Mo_3_S_13_]^2−^ (e.g. EY/PDha‐*g*‐PAA/TiO_2_), and average hydrogen production rates increased from 0.301 mmol g^−1^ h^−1^ (EY/PDha‐*g*‐PAA/TiO_2_) to 23.9 mmol g^−1^ h^−1^ (EY/PDha‐*g*‐PAA/(TiO_2_/[Mo_3_S_13_]^2−^). The high catalytic activity of EY/PDha‐*g*‐PAA/(TiO_2_/[Mo_3_S_13_]^2−^) system (increase by a factor of ≈79) is assigned to the high activity of the molecular co‐catalyst [Mo_3_S_13_]^2−^ when compared with the rather low hydrogen evolution performance of pure TiO_2_. Catalyst accessibility and surface effects might further contribute to the observed increase and will be studied in more detail.

Finally, previous studies by Streb et al.^[11c]^ indicated that [Mo_3_S_13_]^2−^ shows higher catalytic activity in methanol/water mixtures compared to pure water. Therefore, we also investigated our EY/PDha‐*g*‐PAA/(TiO_2_/[Mo_3_S_13_]^2−^ system in a methanol/water (1:1) mixture under otherwise unchanged conditions. As shown in Figure 4 B, we observe increased H_2_ evolution rates in methanol‐water as well as higher apparent stability (i.e. prolonged reactivity compared to the system in water as exclusive solvent). This is in line with previous studies, which suggested that ligand exchange on [Mo_3_S_13_]^2−^ in water is a major deactivation pathway.^[11c]^ Indeed, our data indicates that [Mo_3_S_13_]^2−^ in water shows promising activity but fast catalyst deactivation because of complete exchange of the terminal disulfides resulting in decreased catalytic activity. In contrast, in methanol‐water mixtures, significantly higher reactivity is achieved by stabilizing highly active catalytic species. We could attribute this enhancement to partial exchange of one or two terminal disulfides with aqua ligands which leads to the formation of more active species. Compared to earlier studies where EY was covalently or electrostatically grafted to the surface of TiO_2_, this work demonstrates that PDha‐based polyampholytic graft copolymers are a simple, tunable, and effective method to achieve stable EY sensitization on TiO_2_ with enhanced activity and stability for light‐driven H_2_ evolution.

## Conclusion

In summary, we report the successful grafting of a tailor‐made polyampholytic graft copolymer to TiO_2_ nanoparticles, which enabled the binding of EY photosensitizer and [Mo_3_S_13_]^2−^ hydrogen evolution cocatalysts. Our results reveal that this is a straightforward approach for the preparation of a tunable and versatile soft matter matrix which can effectively co‐integrate several molecular components relevant for light‐driven catalysis. The main role of the graft copolymer is to provide close proximity and the potential to interact for all individual components. Specifically, we find that the obvious improvement of the light‐driven catalytic activity for hydrogen production was found by immobilizing [Mo_3_S_13_]^2−^ clusters, reaching TONs>500. Our strategy indicates that we can use polyampholytic graft copolymers to improve and regulate different molecular catalytic systems by immobilization on TiO_2_. This strategy introduces a system, which could be valuable for other molecular catalysts, dyes or semiconducting sensitizers.

## Conflict of interest

The authors declare no conflict of interest.

## Supporting information

As a service to our authors and readers, this journal provides supporting information supplied by the authors. Such materials are peer reviewed and may be re‐organized for online delivery, but are not copy‐edited or typeset. Technical support issues arising from supporting information (other than missing files) should be addressed to the authors.

Supporting Information
