# Supplementary material for: Polyampholytic Graft Copolymers as Matrix for TiO2/Eosin Y/[Mo3S13]2− Hybrid Materials and Light‐Driven Catalysis
Source: Chemistry. 2021 Mar 8;27(68):16924–9. doi: 10.1002/chem.202100091 (PMC9290844; doi:10.1002/chem.202100091)
Supplement: Supplementary file 1 — Supporting Information [file CHEM-27-16924-s001.pdf]

Chemistry–A European Journal

Supporting Information

Polyampholytic Graft Copolymers as Matrix for TiO_2/Eosin Y/$[Mo_3S_{13}]^{2-}$ Hybrid Materials and Light-Driven Catalysis

Afshin Nabiyan,[a, b, c] Johannes Bernhard Max,[a, b, c] Christof Neumann,[b, c, d] Magdalena Heiland,[e] Andrey Turchanin,[b, c, d] Carsten Streb,[e] and Felix Helmut Schacher*[a, b, c]

Experimental Part

Chemicals

Diethyl vinylphosphonate (>98 %) was purchased from TCI Chemicals and acryloyl chloride (96 %) from Alfa Aesar, ammonium polysulfide solution from Fisher Chemical™ and triethyl amine (≥ 99.0 %) from CHEMSOLUTE®. Triethanolamine (98 %), titanium (IV) oxide (≥99.5 %, 21 nm primary particle size (TEM)), bromotrimethylsilan (97%) and propylamine (98 %), Eosin Y disodium salt (≥85 %) and $(NH_4)_6Mo_7O_{24}*4H_2O$ (99.98 %) were purchased from Sigma-Aldrich. All chemicals were used as received.

$PDha_{0.90}$-*stat*-$PAMA_{0.10}$ was synthesized as described earlier starting from P*t*BAMA with a molecular weight of M_n = 13 200 g/mol and Đ = 2.55 (THF SEC, PS calibration).[1]

Analytical methods

Nuclear magnetic resonance (NMR) spectroscopy

^{1}H- and ^{13}C-NMR spectra were performed on a Bruker AC 300 MHz using $CDCl_3$, MeOD-d_4 and D_2O/NaOD as solvents at a temperature of 298 K. The spectra were referenced by using the residual signal of the deuterated solvent.

Size exclusion chromatography (SEC)

Size exclusion chromatography (SEC) measurements were made on a Shimadzu system equipped with a G1330B pump, a G1362A refractive index detector, a DAD G1315D UV-vis detector and a Polymer Standards Service GmbH (Mainz, Germany) SDV guard/100/1000/100 000 °A, 5 mm column (8 x 300 mm). As eluent THF was used with a flow rate of 1 mL/min. The temperature of the column oven was set to 30 °C and a calibration with low dispersity PS standards was used (M_n ranging from 474 to 2 520 000 g/mol).

SEC measurements in DMSO were performed on a Jasco instrument using DMSO + 0.5 % LiBr as solvent at a flow rate of 0.5 ml/min at 65 °C and Pullulan calibration. It was equipped with PSS NOVEMA 3000 Angsröm / 300 Angström columns, a RI-930 detector as well as a PU-980 pump.

Dynamic Light Scattering (DLS)

DLS measurements were performed using an ALV laser CGS3 Goniometer equipped with a 633 nm HeNe laser (ALV GmbH, Langen, Germany) at 25 °C and at a detection angle of 90°. The CONTIN analysis of the obtained correlation functions was performed using the ALV 7002 FAST Correlator Software.

Transmission Electron Microscopy (TEM)

TEM images were acquired with a 200 kV FEI Tecnai G2 20 equipped with a 4k x 4k Eagle HS CCD and a 1k x 1k Olympus MegaView camera for overview images.

Laser Microelectrophoresis (Zeta-potential measurements)

Electrophoretic mobilities were measured on a ZetaSizer Nano ZS from Malvern via M3-PALS technique with a laser beam at 633 nm. The detection angle was 13°. The samples were prepared by titration of the polymer in 0.1 M NaOH (0.2 g/L) with 0.1 M HCl and 1 mL of the solution was taken at the desired pH values. The titration and pH detection was performed on a Metrohm 765

Dosimat titrator with a Greisinger electronic GMH3539 digital pH-/mV-electrode with a thermometer.

Adsorption and Desorption Studies

An aqueous Eosin Y solution (0.01 mmol/mL) was added to 2 mg/mL of TiO_2 or PDha-*g*-PAA@TiO_2. The resulting suspension was shaken intensively and kept overnight. The following day the particles were removed by centrifugation (14000 RPM), and the supernatant was investigated by UV–vis spectroscopy. In case of solutions with too high concentration, a 10-fold dilution was carried out. The particles were then washed at least 3 times with 1 mL of solution per washing step to remove unbound dye.

X-ray Photoelectron Spectroscopy (XPS)

XPS was performed using a UHV multiprobe system (Scienta Omicron) with a monochromatic X-ray source (Al K_α) and an electron analyzer (Argus CU) with a 0.6 eV spectral energy resolution. For individual samples, charge compensation during data acquisition was realized by an electron flood gun (NEK150, Staib, Germany) at 6 eV and 50 µA. The spectra were fitted using Voigt functions after background subtraction. The spectra were calibrated using the Si 2p peak at 103.5 eV (SiO_2) and the C 1s peak (C-C, 285.5 eV), respectively.

UV-vis spectroscopy

UV-vis measurements were performed on an Agilent Cary 60 spectrometer in a Hellma quartz glass cuvette with a pathlength of 10 mm at room temperature in solvent. The absorbance was measured in a range from 200 nm to 800 nm in 5 nm steps.

Thermogravimetric analysis (TGA)

TGA measurements were carried out under air flow in a Perkin Elmer TGA800 device by heating from 30 °C to 850 °C with a heating rate of 10 K/min.

Ultrasonication (Ultrasonication Finger)

Ultrasonification was carried out using a Sonic VibraCellVC505 500 Watt Ultrasonic Processor. To reduce high sound, a Sound Abating Enclosure was used.

Light-driven catalysis

The light-driven catalytic hydrogen evolution experiments were performed in a 5 or 10 mL Pyrex flask, the openings of which were sealed with a silicone rubber septum, at ambient temperature and atmospheric pressure. A LED light (with max λ = 530 nm (with ± 50 nm; 281 mW; 330 mA; 3.1 V) in a 3D-printed irradiation reactor with a fan) was used as a visible light source to trigger the catalytic reaction and was positioned 2 cm away from the reactor. In a typical light-driven catalytic experiment, the desirable amount of catalyst was dissolved in 2 mL of a mixed aqueous solution containing 0.5 TEOA, and the system was then bubbled with nitrogen for 20 min to remove the dissolved oxygen and create anaerobic conditions. A 0.2 mL sample of the generated gas was collected intermittently through the septum, and the hydrogen content was analyzed by gas chromatography with a thermal conductivity detector (TCD). The turnover number (TON) is determined as moles of hydrogen produced to initial moles of $[Mo_3S_{13}]^{2-}$ (C: 3.0 µmol/mL as initial amount that was used in formation of $[Mo_3S_{13}]^{2-}$@PDha-*g*-PAA@TiO_2).

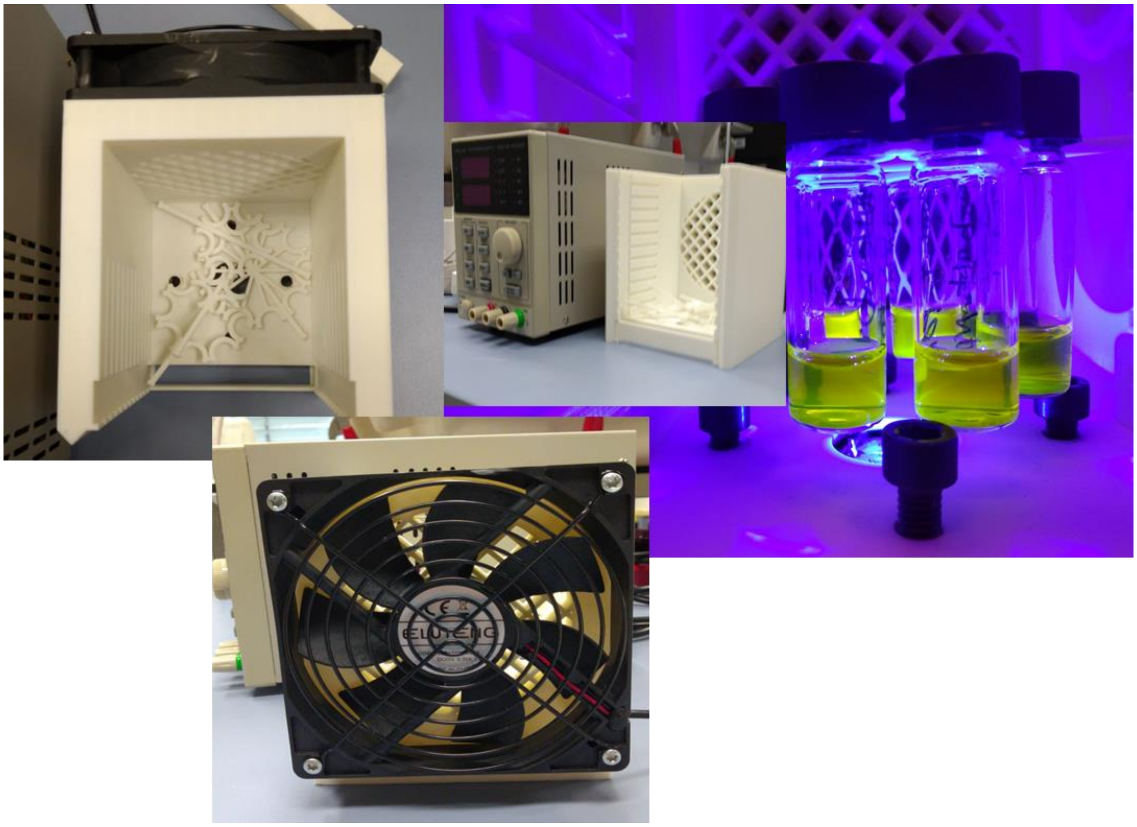

Figure S1: Setup for the light-driven catalytic hydrogen production experiments

Synthesis

Synthesis of PAA in accordance with a literature protocol[2]

N-propyl amine (1.00 g; 17 mmol, 1 eq) and water (20% v.%) were put in an oil bath at 45 °C and afterwards diethyl vinyl phosphate (2.36 g, 14 mmol, 0.85 eq) was added dropwise. The solution was stirred for 18 h before dichloromethane (DCM, ~ 20 ml) was added. Afterwards the reaction mixture was dried with Na_2SO_4, filtrated and the solvent evaporated under vacuum.

The reaction product (1.5 g, 7 mmol, 1 eq) and NEt_3 (1.0 g, 10 mmol, 1.5 eq) were dissolved in distilled DCM (20 ml) and cooled in an ice bath. Acrolyl chloride (0.8 g, 9 mmol, 1.3 eq) was added dropwise and the ice bath removed afterwards. The reaction mixture was stirred for 18 h before it was diluted with more DCM (30 ml). It was washed with 0.1 M NaOH (3 x 30 ml), 0.2 M HCl (2 x 30 ml) and water (2 x 30 mL), dried over Na_2SO_4 and the solvent evaporated under vacuum. The product (1) was obtained as an orange, viscous oil (85 %).

^{1}H NMR (300 MHz, $CDCl_3$, δ) = 6.69 – 6.44 (m, CH_2=CH-), 6.41 – 6.21 (m, -CH=CH_2), 5.70 – 5.58 (m, -CH=CH_2), 4.26 – 3.98 (P-O-CH_2-), 3.76-3.50 (m, -N-CH_2), 3.42 – 3.23 (m, -N-CH_2), 2.25 – 1.89 (m, -CH_2-CH_2-CH_3), 1.76 – 1.52 (m, -CH_2-P=O), 1.45 – 1.19 (m, -CH_2-CH_3), 1.03- 0.80 (m, -CH_2-CH_3) ppm.

Afterwards, the ethyl ester was cleaved. Therefore 1 was dissolved in anhydrous DCM under Ar atmosphere and TMSBr added through a syringe. The reaction mixture was stirred at 21 °C for 24 h. Remaining TMSBr and DCM were removed *via* evaporation and methanol was added. Then

the reaction mixture was stirred at 21°C for another 24 h. After drying under vacuum for several hours the product was obtained as an orange, waxy solid (90%).

^{1}H NMR (300 MHz, MeOD, δ) = 6.76 – 6.56 (m, CH_2=CH-), 6.23 – 5.96 (m, -CH=CH_2), 5.82 – 5.54 (m, -CH=CH_2), 3.35 - 3.20 (m, -N-CH_2), 3.39 – 3.19 (m, -N-CH_2), 1.96 – 1.72 (m, -CH_2-CH_2-CH_3), 1.60 – 1.33 (m, -CH_2-P=O), 0-94 - 0.70 (m, -CH_2-CH_3) ppm.

Post-polymerization modification of PDha-*co*-PAMA with PAA

$PDha_{0.85}$-*stat*-$PAMA_{0.15}$ (100 mg) and PAA (10 eq per monomer unit) were dissolved in water (5 ml each, pH 13, KOH). Afterwards the clear solutions were mixed and placed in an oil bath at 60 °C for constant stirring. The reaction was terminated after 48 h, by adding aqueous HCl (0.5 M) until a pH of 7 was reached. Then, the crude product was dialyzed against deionized water (MWCO = 3.5 kDa) for 2 days and afterwards freeze-dried to obtain a colorless polymer powder.

PDha-*co*-PAMA (50 mg) was dissolved in water (10 ml, pH 13 KOH-solution) and PAA (5 eq per monomer units) was in DMSO (10 ml). The reaction mixture was stirred for 72 h at 70 °C and then it was neutralized using aqueous HCl (0.5 M). For purification, the crude product was dialyzed against deionized water (MWCO = 3.5 kDa) for 48 h and afterwards freeze-dried to obtain a slightly orange powder.

^{1}H-NMR (300 MHz, D_2O, δ) = 3.64 – 3.02 (-CH_2-N-C=O- and (-CH_2-N-C=O-) 2.94 – 2.08 (-C-CH_2-, -NH-CH_2-, -CH_2-CON-) 2.07 – 1.32 (-CH_2-CH_3 and -CH_2-PO_3H_2) 1.29 – 1.07 (-NH- or -$NH_{2)}$, 1.00 - 0.49 (-CH_3) ppm.

^{13}C-NMR (75 MHz, D_2O δ): 177.59 (-COOH), 171.94 (-CONH-), 62.53 - 60.25 (-HN-C-COOH), 49.81, 30.74, 27.28, 26.23, 21.37, 20.28 (side-chain: (-CH_2-NH, -CH_2- PO_3H_2, -CH_2-N-C=O, -CH_2-N-C=O, -CH_2-CON-, -CH_2-CH_3), 45.50 – 42.20 (-C-CH_2-), 10.50 (-CH_3) ppm.

Adsorption study of Eosin Y

An aqueous Eosin Y solution (0.01 mmol/mL) was added to 1 mg/mL of TiO_2 or its compositions that were separated by centrifugation from solution. The resulting supernatant was investigated by UV–vis spectroscopy. In case of solutions that were too concentrated, a 10-fold dilution was carried out. The particles were then washed at least 3 times with 1 mL of solution per washing step to remove unbound dye.

$(NH_4)_2[Mo_3S_{13}]$*$2H_2O$ was synthesized according to the literature.[3] Briefly, to a solution of $(NH_4)_6Mo_7O_{24}$*$4H_2O$ (4.0 g, 3.2 mmol in 20 mL water) a ammonium polysulfide solution (120 ml, 25 wt%) was added. The reaction mixture was covered and heated to 96°C without stirring for five days. Dark red crystals were isolated by filtration, washed with water, ethanol, carbon disulfide and ether before air drying. Yield: 5.6 g, dark red crystals (97.9% based on Mo).

Preparation of PDha-*g*-PAA@TiO_2

The desired amount of polymer was added to the target concentration of TiO_2 in water. Afterwards the mixture was placed in an ice bath. Dispersions were then formed by using an ultrasonic finger (20% power, 60 min, pulsed: 60 s on and 30 s off). For purification, the obtained core-shell hybrid solutions were dialyzed against deionized water (MWCO = 14 kDa) for 48 h.

Preparation and loading of $[Mo_3S_{13}]^{2-}$ in PDha-*g*-PAA@TiO_2

0.5 mg $[Mo_3S_{13}]^{2-}$ were first dispersed with aqueous PDha-*g*-PAA solution (1 mg/mL) with assistance of sonication (1 hour). Afterwards, the stabilized $[Mo_3S_{13}]^{2-}$/PDha-*g*-PAA dispersion was grafted on TiO_2 nanoparticles (2 mg.mL). The grafting process was carried out by 1-hour additional sonication. For purification, the obtained hybrid solutions were dialyzed against deionized water (MWCO = 14 kDa) for 24 h.

XPS analysis

The different preparation steps were characterized using XPS. Besides the Ti 2p spectra discussed in the main paper, the TiO_2 particles show a characteristic feature at a binding energy of ~530.3 eV in the O 1s spectra, which is not present for PDha-*g*-PAA alone (Figure S6). After grafting of PDha-*g*-PAA on TiO_2, the characteristic groups of the polymer are visible in the C 1s spectra as shoulders at binding energies of ~286.6 eV (C-N, C-P) and ~ 288.5 eV (COOH, N-C=O) confirming the presence of PDha-*g*-PAA (Figure S7). Furthermore, P 2p (Figure 3) and N 1s (Figure S7) signals were detected, which were not present on the pure TiO_2 particles before. Finally, the structure of the co-catalyst Mo_3S_{13} was characterized. The Mo 3d signal shows mainly a doublet assigned to Mo_3S_{13} together with a small second doublet ascribed to oxidized Mo. The S 2p signal shows the typical structure for Mo_3S_{13} with two species assigned to bridge/apical and terminal S, respectively. This is in agreement with previously published results.[4]

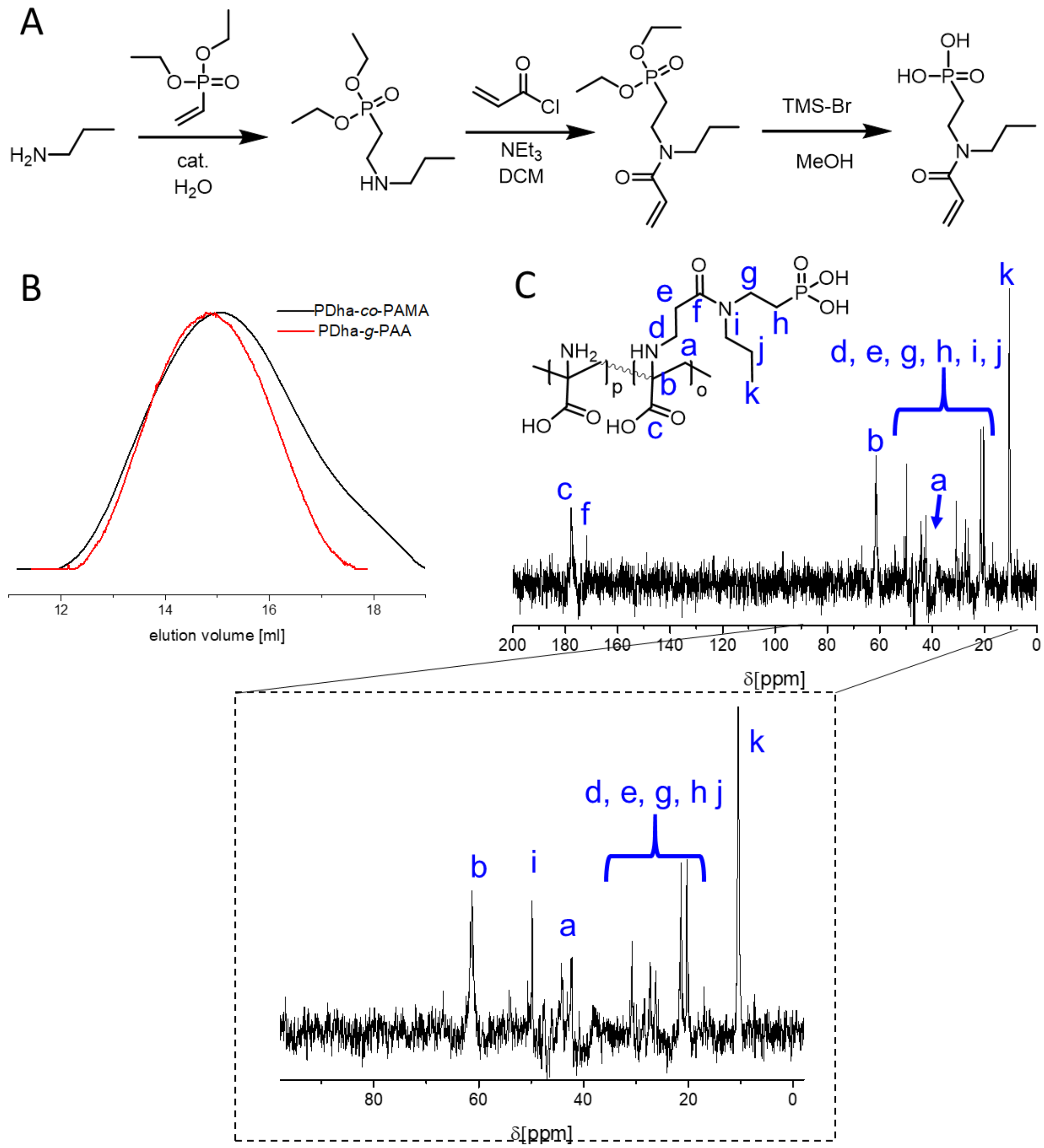

Figure S2: Synthesis of the PAA monomer (A); corresponding SEC trace of PDha-*co*-PAMA and PDha-*g*-PAA (B); ^{13}C NMR spectra of PDha-*g*-PAA (C).

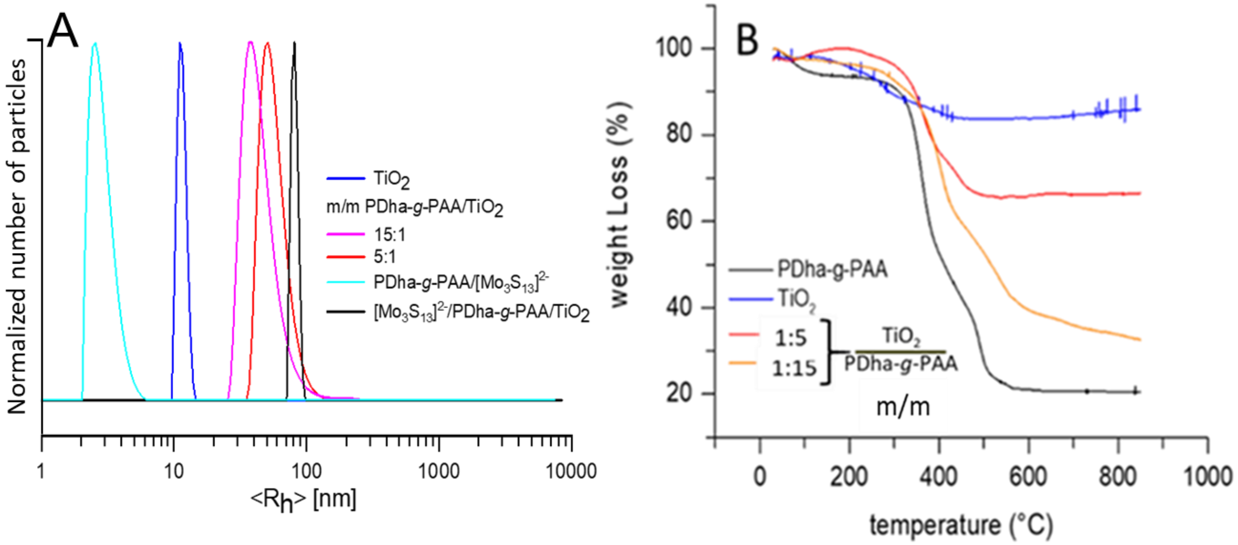

Figure S3: (A): DLS CONTIN plots of TiO_2, PDha-*g*-PAA, PDha-*g*-PAA@ TiO_2, $[Mo_3S_{13}]^{2-}$@PDha-*g*-PAA@TiO_2, and $[Mo_3S_{13}]^{2-}$@PDha-*g*-PAA and (A) and (B): Thermograms of TiO_2, PDha-*g*-PAA, PDha-*g*-PAA@TiO_2 with different ratios of added graft copolymer.

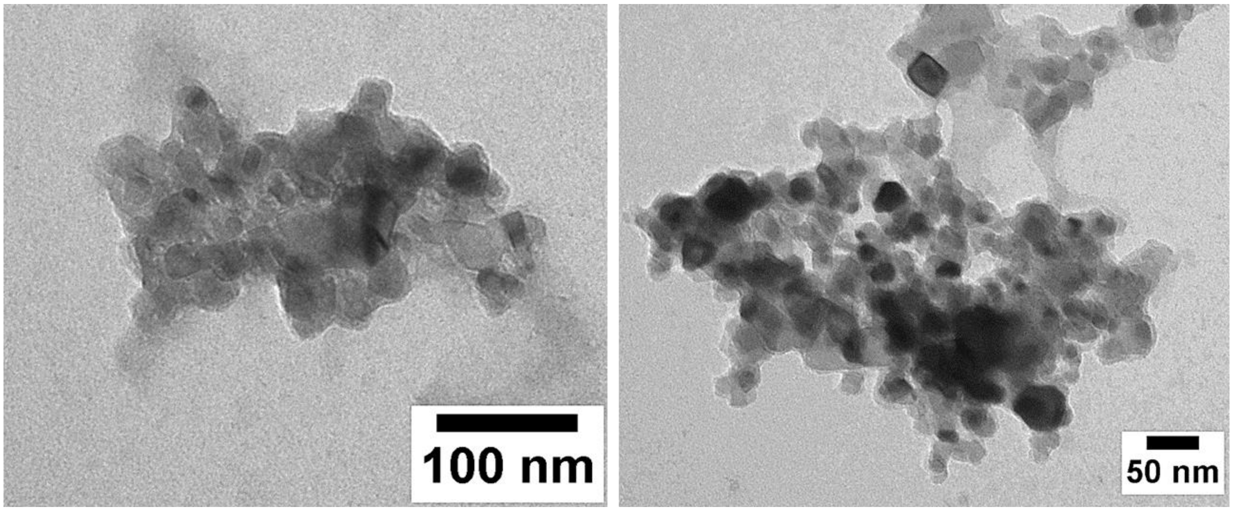

Figure S4: TEM micrographs of PDha-*g*-PAA@TiO_2 with ratio of 5:1 m/m (PDha-*g*-PAA : TiO_2).

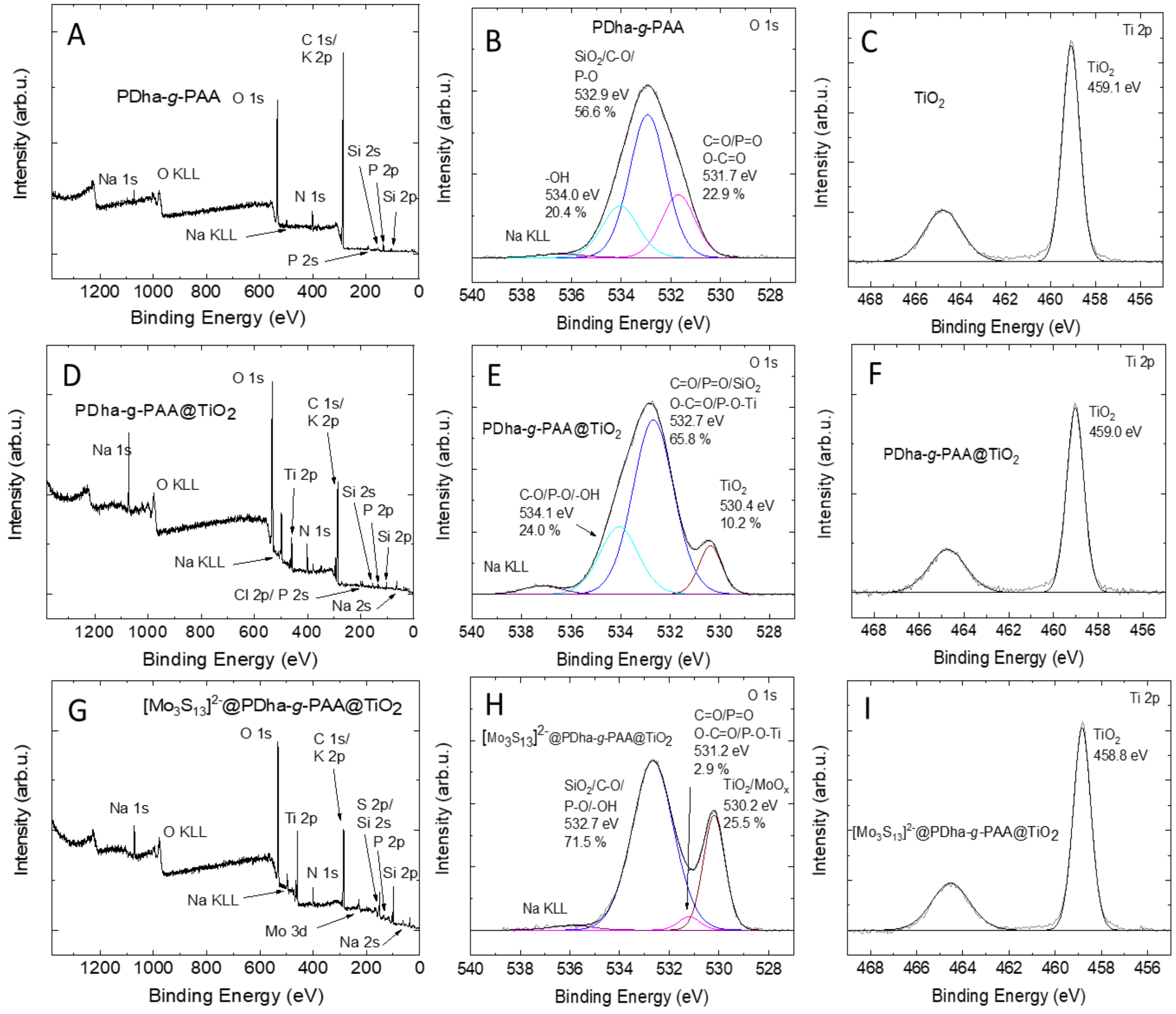

Figure S5: XP spectra of O 1s and Ti 2p of PDha-*g*-PAA, PDha-*g*-PAA@TiO_2 (5:1) and $[Mo_3S_{13}]^{2-}$@PDha-*g*-PAA @TiO_2.

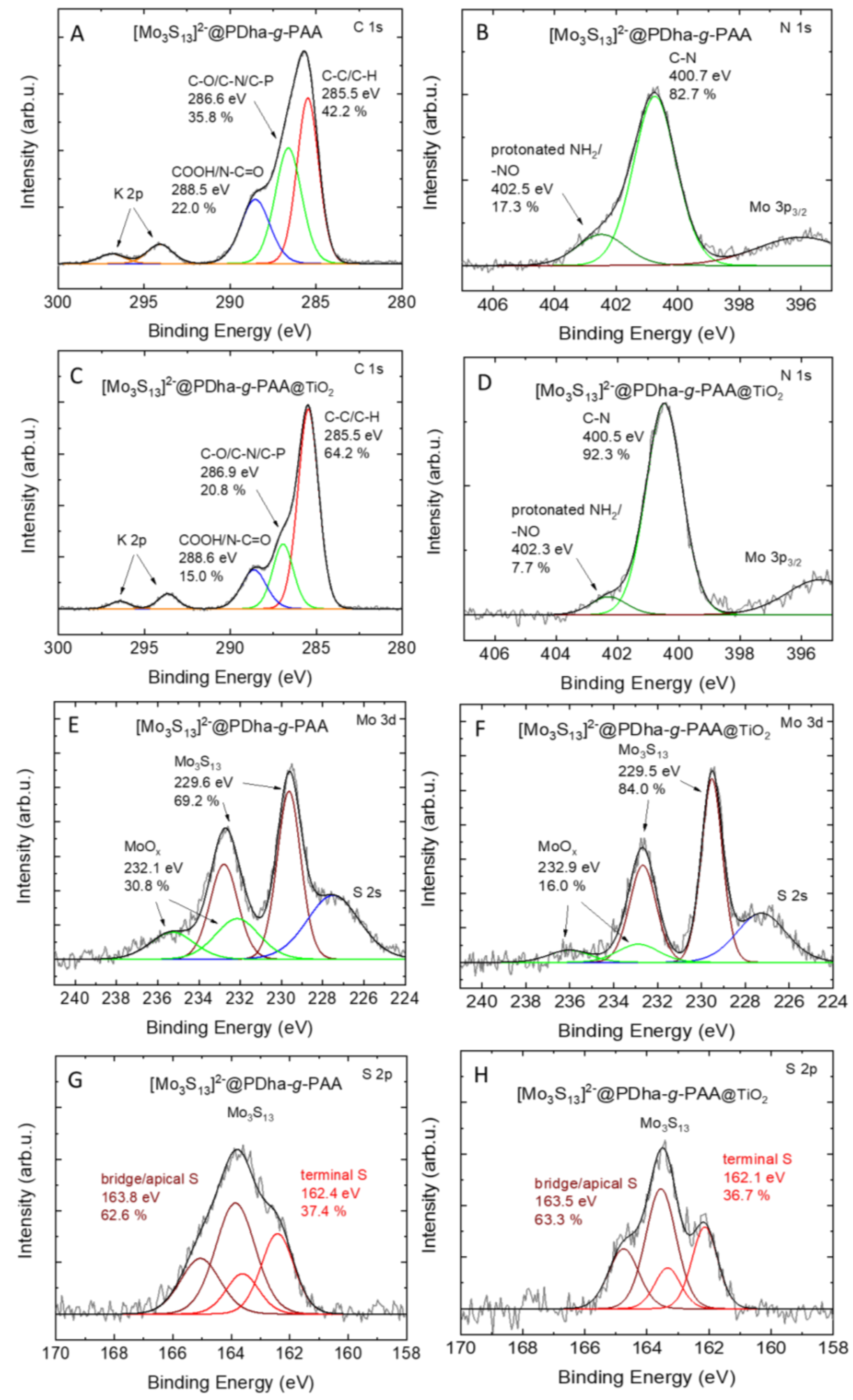

Figure S6: XP spectra of C 1s N 1s, Mo 3d, and S 2p of $[Mo_3S_{13}]^{2-}$@PDha-*g*-PAA and $[Mo_3S_{13}]^{2-}$@PDha-*g*-PAA@TiO_2.

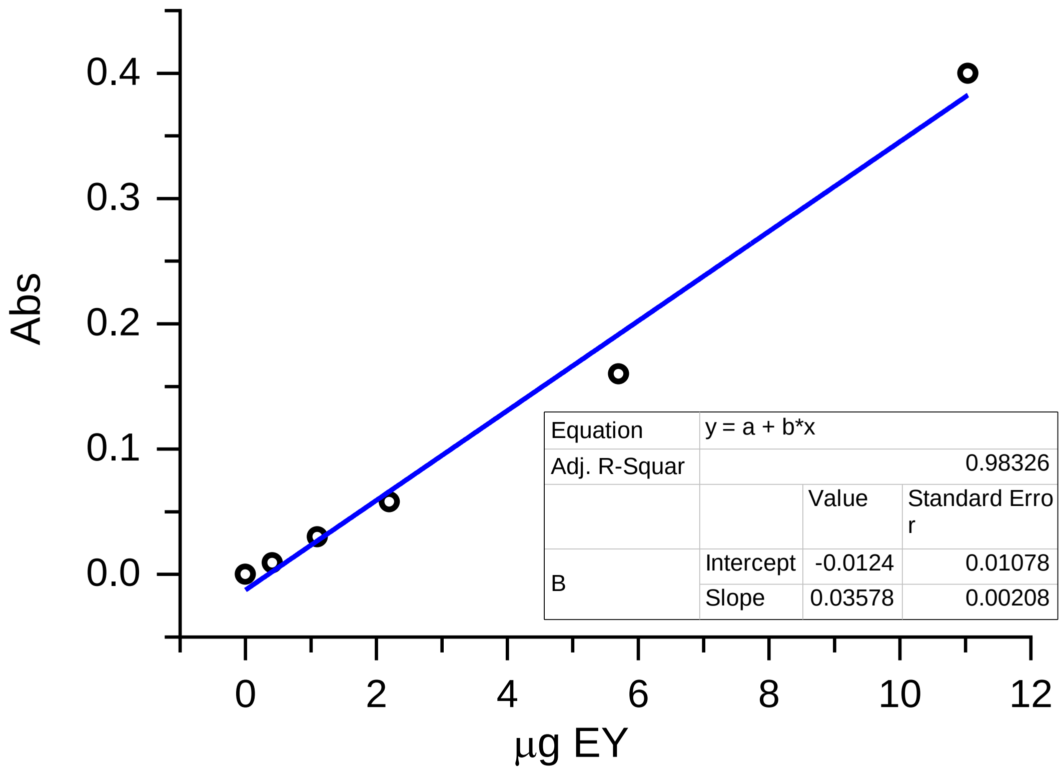

Figure S7: Calibration curves for concentration calculation of EY.

Table S1: Amount of EY absorbed on the different hybrid materials.

**sample**	**Abs 515 nm**	**[a]µg EY[a]**	**µg EY/mg TiO_2**
TiO_2	0.2572	7.53	151
PDha-*g*-PAA@TiO_2 (1:5)	0.1708	5.12	102
PDha-*g*-PAA@TiO_2 (1:15)	0.1237	3.8	76

[a] Calculated after washing the samples with water for 3 times and then re-dispersing by 10 minutes ultra-sonication.

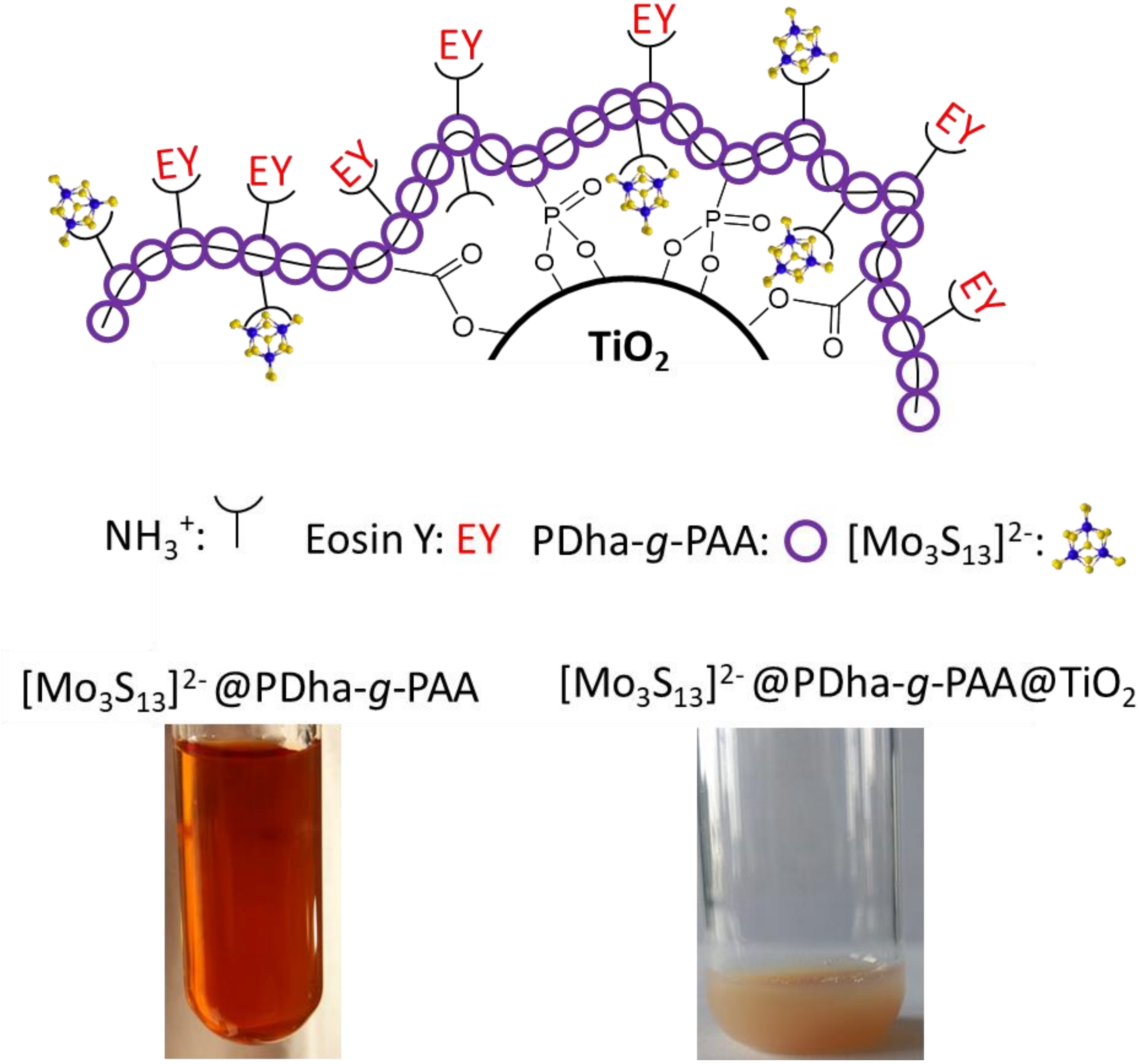

Figure S8: Schematic illustration of proposed (attractive) interactions between PDha-*g*-PAA and TiO_2, Eosin Y, and $[Mo_3S_{13}]^{2-}$.

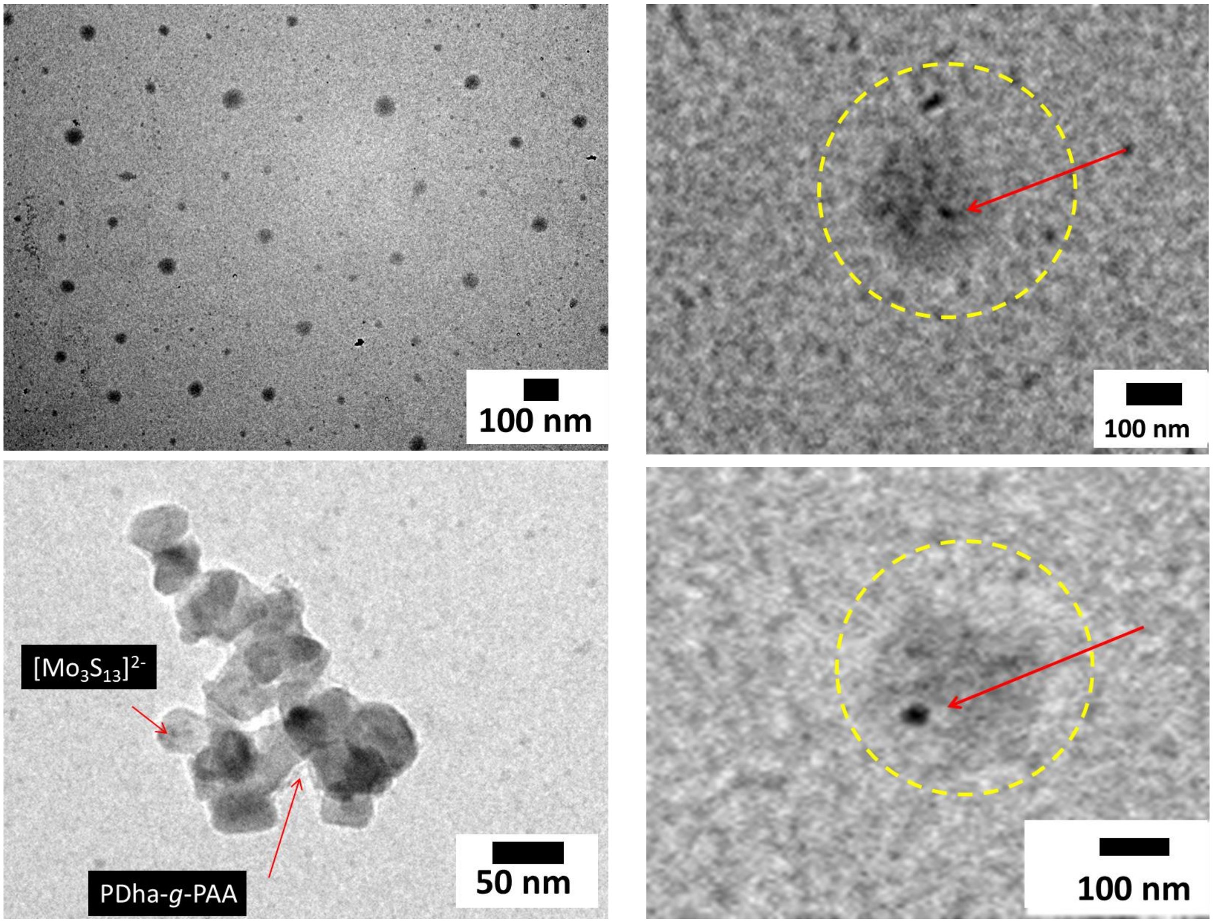

Figure S9: TEM micrographs of $[Mo_3S_{13}]^{2-}$@PDha-*g*-PAA@TiO_2.

References:

1. J. B. Max, K. Kowalczuk, M. Köhler, C. Neumann, F. Pielenz, L. V. Sigolaeva, D. V. Pergushov, A. Turchanin, F. Langenhorst and F. H. Schacher, *Macromolecules*, 2020, **53**, 4511-4523.
2. N. Hu, A. Peralta, S. Roy Choudhury, R. Zhang, R. M. Davis and J. S. Riffle, *Polymer*, 2015, **65**, 124-133.
3. A. Rajagopal, F. Venter, T. Jacob, L. Petermann, S. Rau, S. Tschierlei and C. Streb, *Sustainable Energy & Fuels*, 2019, **3**, 92-95.
4. I. Romanenko, A. Rajagopal, C. Neumann, A. Turchanin, C. Streb and F. H. Schacher, *Journal of Materials Chemistry A*, 2020, **8**, 6238-6244.
